# Temporomandibular disorders in scuba divers: a systematic review

**DOI:** 10.1080/07853890.2021.1897419

**Published:** 2021-09-28

**Authors:** Carla Branco, André Mariz Almeida, Pedro Cebola, Catarina Godinho

**Affiliations:** a Instituto Universitário Egas Moniz (IUEM), Egas Moniz Cooperativa de Ensino Superior, Caparica, Almada, Portugal; b Centro de Investigação Interdisciplinar Egas Moniz (CiiEM), Egas Moniz Cooperativa de Ensino Superior, Caparica, Portugal

## Abstract

**Introduction:**

The number of divers has grown a lot in recent years [1]. The characteristics of the equipment that the divers use in the oral cavity to be able to breathe during the immersion are susceptible to provoke temporomandibular disorders (TMDs) [2]. These patients have specific characteristics related to difficulties in the temporomandibular joint (TMJ), in the masticatory muscles and tissues of the oral cavity. All these complaints are known as "Diver's Mouth Syndrome" [3]. The objective of this study was to obtain what’s known about TMDs in scuba diving.

**Materials and methods:**

Pubmed, cochcrane and B-on were used with the keywords "Temporomandibular Disorders" Mesh Term, AND/OR "Scuba Divers". Studies published in Portuguese, Spanish and English between 2018-1998 on humans were included. We included all clinical trials.

**Results:**

We found 6543 citations of which 2238 duplications were excluded. After screening based on title and abstract analysis, we arrive on 62 full-text articles to assess and selected 8 studies. In terms of study characteristics they can be divided in TMD caused by lack of experience or lack of training in scuba diving [3]; history of TMD previous to scuba diving [4,5]; temperature of the water and facial pain [5]; and TMD and design, material and universal or customised mouthpiece (MP) and TMD [5–8]. **Discussion and conclusions:** Diving in colder waters is related with an increase in facial pain due to muscle contraction and clenching of masticatory muscles thus inducing facial pain [5]. In terms of TMD and MP for oxygen bottles the major part of the studies reveal the more customised the MP the less prone to TMD the subject is, so we have the standard MP, less efficient assuming that the same measure is for all mouths; the temperature mouldable MP to the mouth after being softened in hot water which deform with ease and the customised MP that are more effective, more expensive and constructed in the likeness of each one by the dentists so recognised by the sleep society [5]. Inexperienced divers tend to be more prone to TMD due to the mistake they make and stress they are exposed to. Due to the study design being so weak until now more studies should be carried out on the clinical side in a standardised way so that studies can be compared rather than based only on self-report.
